# Novel MRI-Based CAD System for Early Detection of Thyroid Cancer Using Multi-Input CNN

**DOI:** 10.3390/s21113878

**Published:** 2021-06-04

**Authors:** Ahmed Naglah, Fahmi Khalifa, Reem Khaled, Ahmed Abdel Khalek Abdel Razek, Mohammad Ghazal, Guruprasad Giridharan, Ayman El-Baz

**Affiliations:** 1 Department of Bioengineering, University of Louisville, Louisville, KY 40292, USA; ahmed.naglah@louisville.edu (A.N.); fakhal01@louisville.edu (F.K.); guruprasad.giridharan@louisville.edu (G.G.); 2 Faculty of Medicine, Mansoura University, Mansoura 35516, Egypt; reemkhaled@mans.edu.eg (R.K.); arazek@mans.edu.eg (A.A.K.A.R.); 3 Electrical and Computer Engineering Department, Abu Dhabi University, Abu Dhabi 59911, United Arab Emirates; mohammed.ghazal@adu.ac.ae

**Keywords:** thyroid, cancer, CNN, MRI, DWI, radiomics

## Abstract

Early detection of thyroid nodules can greatly contribute to the prediction of cancer burdening and the steering of personalized management. We propose a novel multimodal MRI-based computer-aided diagnosis (CAD) system that differentiates malignant from benign thyroid nodules. The proposed CAD is based on a novel convolutional neural network (CNN)-based texture learning architecture. The main contribution of our system is three-fold. Firstly, our system is the first of its kind to combine T2-weighted MRI and apparent diffusion coefficient (ADC) maps using a CNN to model thyroid cancer. Secondly, it learns independent texture features for each input, giving it more advanced capabilities to simultaneously extract complex texture patterns from both modalities. Finally, the proposed system uses multiple channels for each input to combine multiple scans collected into the deep learning process using different values of the configurable diffusion gradient coefficient. Accordingly, the proposed system would enable the learning of more advanced radiomics with an additional advantage of visualizing the texture patterns after learning. We evaluated the proposed system using data collected from a cohort of 49 patients with pathologically proven thyroid nodules. The accuracy of the proposed system has also been compared against recent CNN models as well as multiple machine learning (ML) frameworks that use hand-crafted features. Our system achieved the highest performance among all compared methods with a diagnostic accuracy of 0.87, specificity of 0.97, and sensitivity of 0.69. The results suggest that texture features extracted using deep learning can contribute to the protocols of cancer diagnosis and treatment and can lead to the advancement of precision medicine.

## 1. Introduction

In the United States, approximately 52,890 new cases of thyroid cancer and about 2180 deaths were estimated in 2020 according to the American Cancer Society’s most recent statistics [1]. The prevalence of thyroid nodules is approximately 5% in women and 1% in men [2]. Among the cases of thyroid nodules, 7–15% evolve into malignant tumors (cancerous tissue), and this rate depends on age, sex, radiation exposure history, family history, and other factors [2]. Malignant tumors can be classified into three major categories: Differentiated thyroid cancer (DTC), medullary thyroid cancer, and anaplastic thyroid cancer. DTC has the biggest share of thyroid cancer, with a share of more than 90%. DTC includes two main subcategories: papillary thyroid carcinoma (PTC) and follicular thyroid carcinoma (FTC). PTC accounts for more than 80% of all thyroid cancers [2].

The diagnostic criteria of thyroid nodules involve different procedures that include physical examination, blood test, ultrasound (US) imaging, magnetic resonance imaging (MRI), and a biopsy procedure. The detection of smaller nodules becomes easier over time due to the current advances in US and MRI. However, cancer diagnosis and early stratification of nodules is still challenging and mainly performed using biopsy [2]. Although biopsy, either fine-needle aspiration or surgical excision of the nodule, is still the definitive way of clinical evaluation, this invasive procedure is costly and may introduce a false negative error depending on the biopsy technique and the size of the nodule being aspirated [3,4,5,6].

Non-invasive-based approaches have been proposed by several researchers to provide accurate detection and stratification of thyroid cancer [7,8,9,10]. These methods utilize different types of medical images. The type of imaging technology used as an input to artificial intelligence (AI) algorithms can affects the accuracy of the desired computer-aided diagnosis (CAD) system. US imaging is currently used as a first-line evaluation of suspected thyroid nodules [2], and specific features of thyroid nodules in US imaging can be associated with higher risk of malignancy. However, the appearance of those features in US images is operator-dependent, and also multiple features need to be considered simultaneously during the evaluation in order to provide sufficient malignancy diagnostic power [2]. These factors cause various limitations in AI-based systems that use US images for thyroid nodule classification [7,8,9]. Compared to US, MR imaging modalities have also been used in the literature recently. For instance, T1-weighted MRI and T2-weighted MRI were used in a recent study to perform thyroid nodule classification [10]. Some MRI modalities can help distinguish between different substances in the tissue. For example, fats appear bright in T1-weighted MRI images [11], while fluids appear bright in T2-weighted MRI images. Studying T2-weighted MRI images can help in the modeling of fluid patterns in the tissue [12]. Over and above that, diffusion-weighted MRI (DWI) can model the diffusivity of fluids in the tissue by measuring constraints of fluid diffusion in different directions [13,14]. Therefore, DWI can model the dynamics of fluids in the tissue, and these dynamics can be presented by computing the apparent diffusion coefficient (ADC).

**Figure 1:**
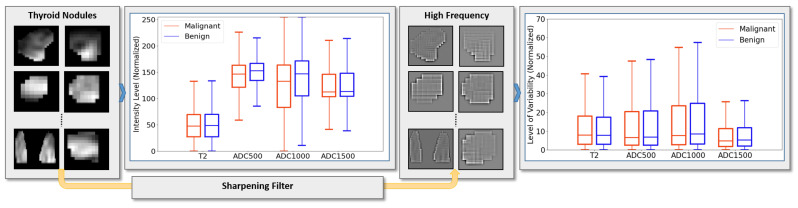
Illustrative diagram of the preliminary statistical study performed on our dataset. A high-pass Laplacian spacial filter was applied to the images to estimate intensity variation at the pixel level. Following that, statistical analysis was performed to calculate the mean difference between malignant and benign nodules.

The cell proliferation process associated with malignant thyroid nodules can have a significant effect on the patterns and the dynamics of the extracellular matrix (ECM) in the thyroid tissue. Studies suggest that statistical analysis between ADC value and T2-weighted images, and therefore can differentiate between malignant and benign nodules [15,16,17]. Thus, in the preliminary analysis of our work, we examined if the intensity variations between malignant and benign groups are significantly different or not, see Figure 1. To achieve this, we employed a statistical analysis test to determine the differences between the two groups as observed in each of the T2-weighted images and the ADC maps (three different gradient coefficients were used to generate the ADC maps). Our analysis showed significant heterogeneity in intensity variance between T2-weighted images and ADC maps, which suggests that feeding the T2-weighted images and the ADC maps each to a separate input branch of the CNN would enables learning of independent textures in each branch and therefore this would enhance the accuracy of our system.

**Figure 2:**
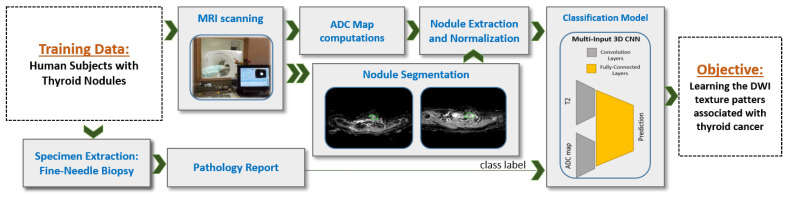
Schematic diagram that represents the training pipeline for the proposed system. MRI data were collected from human subject cohort. ADC maps were computed in order to prepare the two inputs for the CNN. The objective of the proposed system was to learn the texture patterns in DWI images and correlate them with pathological finding.

Inspired by our preliminary statistical analysis results, our initial exploratory work [18], and other studies [15,16,17], we propose a novel CNN-based CAD system that integrate T2-weighted images and ADC maps using a multi-input CNN network for thyroid nodules detection and classification, see Figure 2. Our work is in contrast to one recent study that proposed a CNN-based system using multimodel MRI but does not include ADC maps [10]. ADC maps can be considered as an indication of cell density in tissues [19] and therefore can be used to search for cancer biomarkers, which usually involve high rates of cell proliferation. Similar to a recent study [20] that uses multiparametric MRI radiomics for prediction, we use a CNN-based structure instead of hand-crafted features—namely, we utilize a process of independent convolutions for ADC and DWI before fusing them using the dense fully connected layer. This process increases the possibility to detect deep texture patterns from each modality without loosing the capability for automatic searching for visual features, provided by the CNN. Our system integrates multiple ADC maps obtained from different gradient coefficients (a configurable parameter in the MRI scanner) for each sample. Then, the combination of all inputs is fed to our CNN model as a multichannel 3D input in order to achieve enhanced learning of texture features, thus providing a more accurate diagnosis.

## 2. Materials and Methods

### 2.1. Study Participants and Data Collection

Data were collected in this study from 49 patients with pathologically proven thyroid nodules. The age range is 25 to 70 years. Imaging of the thyroid gland was performed at Mansoura University, Egypt with a 1.5 T Ingenia MR scanner (Philips Medical Systems, Best, Netherlands) using a head/neck circular polarization surface coil. All participants were fully informed about the aims of the study and provided their informed consent. The inclusion criteria for the study were untreated patients with thyroid nodules whose malignancy status was unclear from ultrasound examination. Patients underwent thyroid core biopsy or surgery after MR imaging. Histopathologic diagnoses were provided by an experienced cytologist or pathologist. In total, there are 17 malignant nodules in 17 patients and 40 benign nodules in 32 patients included in our study.

DWI volumes that employ a multislice, single-shot, spin-echo, echo-planar imaging sequence with TR = 10,000 ms, TE = 108 ms, and 125 kHz bandwidth were extracted. Axial diffusion-weighted slices over the region of interest were 5 mm thick with an inter-slice gap of 1 mm, 25 cm or 30 cm FOV, and 256 × 256 acquisition matrix. For DWI, a diffusion gradient was applied during scanning with *b*-values of b=500 s/mm${}^{2}$, b=1000 s/mm${}^{2}$, and b=1500 s/mm${}^{2}$. T2-weighted images are extracted using b-value of b=0 s/mm${}^{2}$.

### 2.2. ADC Map Calculation and Nodule Segmentation

Multiple steps were applied to the collected MR images in order to prepare the dataset to be used by the training model, see Figure 2. Nodule segmentation was performed manually in our study. An experienced radiologist segmented each nodule as it appeared in each T2-weighted slice (b=0 s/mm${}^{2}$) and in each DWI slice. Diffusion-weighted MRI scans were taken in the same session and using the same resolution, number of slices, and inter-slice gap. Therefore, no registration was applied to align the different *b*-values. We have future plans to implement an automated segmentation algorithm for nodule extraction. The produced manual segmentation was stored in the form of binary images. The binary image produced from DWI slice with b=0 s/mm${}^{2}$ was re-used during processing phases on the corresponding slice at all other *b*-values, and also was re-used for the corresponding slice at ADC500, ADC1000, and ADC1500. We extracted each nodule in both T2-weighted images and ADC maps using a square-bounding box. We regularized the spatial domain by resizing extracted box into unified 48 × 48 × 20 volumes by adding zero-padding channels. We then normalized the voxel-intensity in that volume to be in 0–1 range. Each segmented nodule was provided for the network model on a black background and padding. Apparent diffusion coefficients (ADC maps) were calculated at each non-zero b-value (500, 1000, and 1500) by combining the diffusion images at the corresponding b-value with the image at b=0 s/mm${}^{2}$, and then we substituted, at the voxel level, this into the Stejskal–Tanner equation [21]. The generated images of this process are referred to as ADC500, ADC1000, and ADC1500. Since diffusion-weighted MRI (DW-MRI) as an absolute value usually does not reflect direct biological activity, the relative differences between DW-MRI at different *b*-values were used instead (i.e., ADC) to model the diffusivity in the tissue. Usually, a *b*-value of 0 is taken as reference, and which is why we computed three ADC values that correspond to 3 *b*-values of 500, 1000, and 1500 referenced to a *b*-value of 0.

### 2.3. Proposed Learning Model: Multi-Input CNN

**Figure 3:**
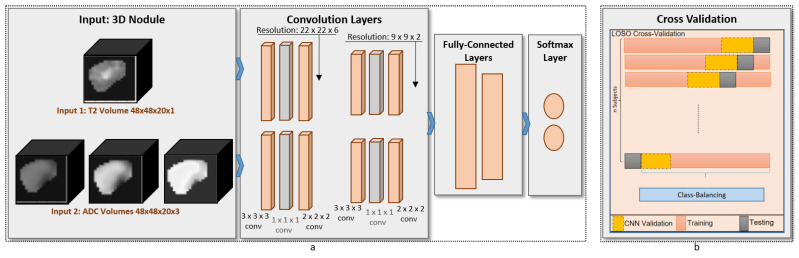
(**a**) Schematic diagram of the proposed CAD system that shows the design and the layers of the multi-input 3D CNN deep-learning framework. (**b**) Illustrative diagram that shows the cross-validation criteria used in our processing.

To build our diagnostic system, we propose a novel multi-input deep-learning network. Our architecture follows the feed-forward convolutional neural network (CNN) structure. Our implementation uses the Keras package in Python, and the parameters used in our training model are summarized in Table 1. The proposed architecture, shown in Figure 3, consists of two identical branches in the structure. The advantages of our network compared with others is that the generated kernels are governed by the fusion of T2-weighted images and ADC maps of the training samples during the forward propagation and backward propagation of our neural network. Additionally, a 1×1×1 *3Dconv* layer was added to the proposed design in order to perform compression for the features maps. The advantage of this addition is that the number of weights that needs to be learned during the training phase is extremely minmized, thus ensuring fast learning and diagnosis. For the analysis, each of the base images and the ADC maps was fed to the respective branch. The convolution layers were constructed from 3×3×3 *3Dconv* (with 32 filters and 3×3×3 kernel size), 1 × 1 × 1 *3Dconv* (with 16 filters and 1×1×1 kernel size), pooling block (2×2×1 pool size, maximum value pooling). Each branch had two convolution blocks before being concatenated into the dense fully connected layers (2 layers). Those layers were one hidden layer of 10 neurons with ReLU activation function [22] and one output layer of 1 neuron with sigmoid activation function [23]. The total number of parameters in our proposed network is 127,829 parameters.

The condition of unbalanced classes during the training phase was handled by configuring the weights in the mean-square error (MSE) loss function we used in the back propagation of the network. The ratio of the weight of malignant class to the weight of benign class was set to 16/32 when leaving out one malignant sample for testing, and the same ratio was set to 17/31 when leaving-out one benign sample for testing. The loss function used is given in Equation (1), where *N* is the number of training samples, *y* is the output of the neural network observed during forward propagation, $y_{i}$ is the label of the sample, and $w_{i}$ is the weight of each training sample.

(1) $$Loss=\frac{1}{N}\sum_{i=0}^{N}w_{i}{\left(y-y_{i}\right)}^{2}$$

We used Adam stochastic to update the parameters of the network during learning [24]. The learning rate and other parameters of the optimizer were tuned and kept constant during our evaluation. Additionally, we used the ratio of 1 to 3 of the samples as validation data during the learning phase.

### 2.4. Other Learning Models

In order to perform bench-marking for our system, we compared its performance with other methods. We first compared the results with ML methods that use hand-crafted features, and then we compared the results with two state-of-the-art CNN models. Regarding the first comparison, the used hand-crafted features can be classified into three groups: shape features, statistical features, and hand-crafted texture patterns features. Starting with the shape features, we used nodule size (in voxels), convex hull ratio (defined as the ratio between the nodule size and the convex hull size), bounding rectangle ratio (defined as the ratio between the nodule size and the bounding rectangle size), and spherical harmonics of 3D contour encapsulating the nodules. We estimated the spherical harmonics inspired by [25] by the use of infinite set of harmonic functions defined on a spherical representation. They arise from solving the angular portion of Laplace’s equation in spherical coordinates using separation of variables. The degree of the spherical harmonics can define the level of non-homogeneity of the surface, and we can map this to the ability to differentiate between malignant and benign nodules.

For the statistical features, we calculated the histogram of each image, and then in each histogram we summarized their statistical profile using 5 features (mean, standard deviation, entropy, skewness, kurtosis). This type of features is designed to summarize the whole image by presenting it using certain values. The overall appearance of thyroid nodule can reflect the first impression by experienced radiologists while examining the MRI scan. Finally, for the hand-crafted texture patterns we built a filter-bank of 9 filters to evaluate intensity variations between neighbor voxels. The used filter-bank is designed to capture edges in 4 orientations, lines in 4 orientations, and the point response (all-directions variability). The four orientations are horizontal, vertical and 2 diagonal orientations.

All features from the three hand-crafted features groups were evaluated for malignancy detection capability using four different classifiers: decision tree (DT) [26], random forest (RF) [27], Naive Bayes (NB) [28] and support vector machine (SVM) [29]. The classification models used in the benchmark were optimized to ensure appropriate comparison. In DT, min sample split was examined. In RF, number of estimators and maximum depth were examined. In SVM, C parameter is examined to tune the soft margin.

In addition to traditional ML methods, we compared our methods accuracy against other CNN-based methods. For bench marking purpose, we used two state-of-the-art CNN models for detection; AlexNet [30] and ResNet18 [31]. AlexNet is chosen as it is the first deep learning computer vision to be recognized as a classification-winner of ILSVRC [32] back in 2012. ResNet is chosen because it is the first ILSVRC winner that overachieve human accuracy in classification under different appearance conditions [33]. For both methods, we used Keras implementations in Python with the default configuration. AlexNet and ResNet were applied to the combined T2-ADC input in the form of multiple input channels.

### 2.5. Evaluation Criteria

The evaluation criteria of our system use a leave-one-out cross-validation. We kept the common network configuration fixed for our reported results, including the ablation study, as well as when compared with other techniques. The proposed system evaluation is based on four classification metrics: accuracy, precision, recall, and dice coefficient.

**Figure 4:**
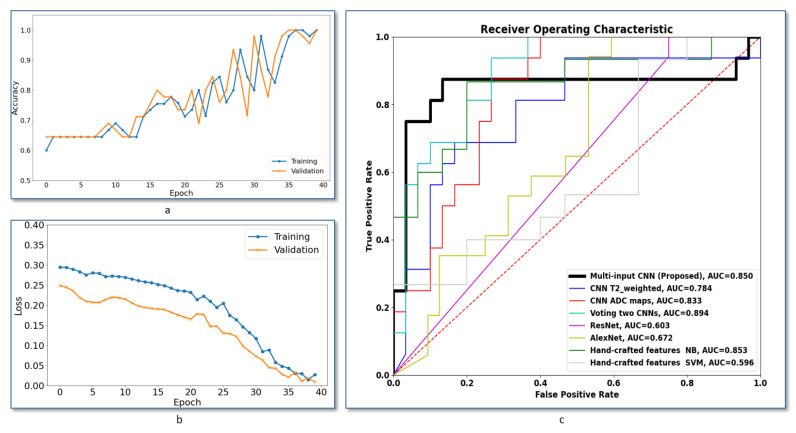
(**a**) Training versus validation accuracy curves with the number of epochs during network training. (**b**) Training versus validation loss curves with the number of epochs during network training. (**c**) Receiver operating characteristic curves (ROCs) of the proposed multi-input CNN framework compared to other methods. AUC is the area under the curve.“DT”—Decision Tree. “RF”—random forest; “NB”—Naive Bayes; “SVM”—support vector machine.

Additionally, further evaluation of the system robustness has been conducted using the the receiver operating characteristics (ROC) analysis curve. The ROC curve is a plot between the false positive rate and the true positive rate when we adjust the decision threshold. Figure 4c shows ROCs of the proposed multi-input CNN framework compared to the other frameworks discussed in this section. The area under the curve (AUC) of the voting between two CNNs gives slightly higher value, but our system achieved the best AUC compared with all other methods.

For the purpose of this analysis, the slice at which each thyroid nodule appears with biggest size was extracted and processed as a 2D image for each of T2-weighted image and ADC maps. Local intensity variations were modeled by high-pass filtering using a 3×3 Laplacian filter invariant to 45${}^{\circ }$ rotations [34]. Tumor pixels were grouped into benign and malignant groups (35,625 and 15,764 pixels, respectively). Supported by the high number of samples, a Welch two-sample *t*-test was applied to determine difference the mean between groups. A statistical package in R was used to generate the results.

### 2.6. Nodule Texture Visualization

Achieved kernels applied to each of the T2-weighted images and the ADC maps were extracted from CNN network after the last epoch of training cycles. The extracted kernels are converted from the 3D to 2D form by averaging the 3 depth channels. The kernels were then clustered using hierarchical agglomerative clustering [35,36]. Silhouette score was used for evaluating the fit of the estimated clusters [37]. The Sklearn package in Python was used for both clustering processing and evaluation.

## 3. Experimental Results

The overall proposed framework is depicted in Figure 3. In this section, we present our results, which include: (1) preliminary statistical analysis, (2) the performance of the proposed CAD system compared to other machine learning models that use hand-crafted features, (3) the performance of the proposed CAD system compared to state-of-the-art CNN models, and (4) the results obtained of analyzing the texture patterns after learning.

### 3.1. Significant Differences in T2 and ADC Local Intensity Variations between Malignant and Benign Groups

The results of analyzing local intensity variations in each of the T2-weighted images and the ADC maps show that there is a significant difference in the mean of those variations between benign and malignant groups. Table 2 presents the results obtained from the Welch two-sample *t*-test that shows a significant difference with p<0.05. Table 2 also presents the achieved *t* value and the 95% confidence interval (CI). The CI values are normalized with respect to the standard deviation (SD) of the benign group. By observing the sign of CI, the malignant group has higher mean observed in T2-weighted images while the benign group has a higher mean in ADC maps. This result suggests that having convolution filters of T2-weighted images that are independent from those of ADC maps enables conducting enhanced texture-learning process. Convolution filters map the conv kernels in our proposed CNN architecture.

### 3.2. Comparison with ML Methods That Use Hand-Crafted Features

The results are summarized in Table 3. As can be seen, the proposed multi-input CNN system outperform all compared classifiers. Our proposed CAD system achieved the best performance when compared to machine learning models that are based on hand-crafted features. Our system achieved an AUC of 0.85 compared to 0.59 when using linear support vector machine (SVM) classifier, see Figure 4c. Additionally, it achieved an accuracy, sensitivity, and specificity of 0.87, 0.69, and 0.97, respectively, compared to an 0.77, 0.67 and 0.77 when using random forest (RF) classifier, which achieved the best accuracy among the pool of classifiers used with hand-crafted features. The results in Table 3 show that using automatic feature selection by the aid of CNN helps in achieving better diagnostic accuracy.

### 3.3. Comparison with State-of-the-Art CNNs

In addition to the comparison with the handcrafted-based ML approaches, comparison against other state of the arts CNN models have been conducted. The comparative results, shown in Table 4 also showed that the proposed CAD system achieved the best diagnostic performance. It is worth mentioning that our system has relatively low number of layers compared to the compared models. It achieved an AUC of 0.85 compared to 0.67 and 0.60 obtained using AlexNet and ResNet 18, respectively. Additionally, it achieved an accuracy of 0.87, sensitivity of 0.69 and specificity of 0.97. The accuracy, sensitivity and specificity using AlexNet were 0.61, 0.53, and 0.66, respectively, and those obtained using ResNet18 are 0.49, 1.00 and 0.22, respectively. Results document that using lower number of CNN layers can achieve better diagnostic accuracy, which is considered an advantage of the proposed method compare with other CNN-based techniques.

### 3.4. Texture Features of T2-Weighted Images Are Visually Different Compared to ADC Maps

**Figure 5:**
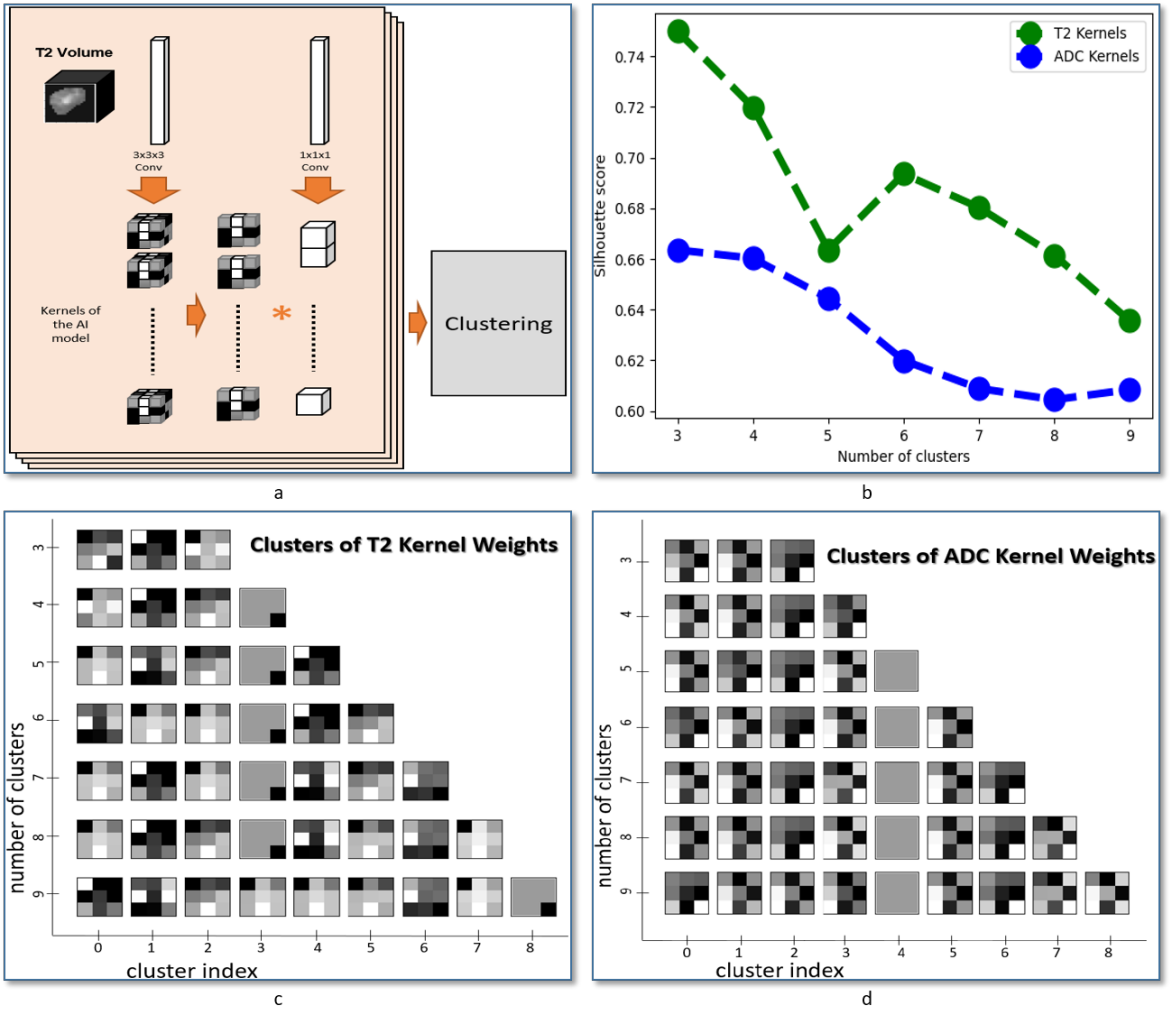
Analysis of the patterns extracted from the CNN after training phase. (**a**) Illustrative diagram of the process of extracting the kernels from the weights of each layer, and the processing of those kernels using a clustering technique (hierarchical agglomerative clustering) in order to analyze the patterns found in T2-weighted MRI images and ADC maps. (**b**) Evaluation metric of the clustering algorithm by computing Silhouette score while varying the number of clusters in the clustering algorithm. (**c**) Visualization of the results of our analysis on the features extracted from T2-weighted images. (**d**) Visualization of the results of our analysis on the features extracted from ADC maps. We can notice that the texture patterns that distinguish between malignant and benign thyroid nodules are having a degree of heterogeneity according to this visualization.

The convolution kernels (filters) extracted from the CNN after learning were clustered, see Figure 5a, and the clustering process was repeated for multiple runs each with different number of target clusters k=2,4,5,...,9. Figure 5b shows the evaluation of the generated clusters using the Silhouette score. The clusters generated from the T2-weighted kernels (green curve) achieved better clusters compared to ADC kernels (blue curve). Additionally, k=3 achieved the highest score in both T2-weighted and ADC images. Figure 5c,d show the visualization of the generated clusters of T2-weighted and ADC kernels, respectively. The runs (with the corresponding number of clusters, or *k*) are represented on the y-axis. Each row includes the generated clusters of the corresponding run, and the cluster index inside each run is presented on the x-axis. Each cluster is illustrated by the mean of its member kernels, and then each mean is normalized from 0 to 1. A gray-scale visualization of each normalized mean is presented (at each row-column position) using a 3×3 board image in a way that 0–1 is mapped to a white–black gradient.

## 4. Discussion and Conclusions

We proposed a new CAD system to distinguish between malignant and benign thyroid nodules. The main contributions of the proposed pipeline is the use of multi-input CNN that can detect texture patterns from each input independently. The first branch of our CNN models the fluids patterns in the thyroid tissue by learning the texture patterns in T2-weighted MRI images. The second branch of our CNN models the dynamics of tissue fluids by learning the texture patterns in ADC maps. We validated our method by applying leave-one-out cross-validation on multimodal data collected from 49 patients with pathologically confirmed nodules. We compared the classification accuracy obtained from our system with other ML and deep learning approaches. Experimental results from our system surpass results obtained from other models.

To assess the advantage of integrating multiple MRI modalities as separate inputs of the proposed network, we conducted a preliminary study that shows heterogeneity in the intensity variation between malignant and benign samples. In this experiment, a Welch two-sample *t*-test was used to assess the significant difference in mean variation between the two groups (Table 2) across all modalities. The difference in mean between the two groups in T2-weighted images has an opposite sign when compared to the corresponding difference in ADC maps (Table 2). This also suggests that using independent features in each input can enable finding more optimal features.

To assess the performance of our system, we compared it to other ML methods that use hand-crafted features. In the comparison, we used three categories of hand-crafted features. The first category is based on the statistical profile of image intensity. We evaluated that statistical profile using five features (mean, standard deviation, entropy, skewness, kurtosis). This category is designed to summarize the whole image by presenting it using the profile of each features. The overall appearance of the tumor can reflect the first impression by the physician while examining the MRI scan. The linear SVM classifier exhibited the worst performance, which suggests a lack of a linear border between classes. Results of the NB classifier showed the possibility of having a fairly distinguished statistical distribution of the hand-crafted features extracted from benign and malignant nodules. In order to benchmark our system, Figure 4c shows ROCs of the proposed multi-input CNN framework compared to the other systems under comparison. As demonstrated, the area under the curve (AUC) of our system is higher compared with all compared methods, which highlights the higher accuracy of our method. Figure 4a,b show the training versus validation accuracy and loss curves during the model training. Overall, the results showed that handcrafted features failed to provide a good modeling of our classification problem, and this suggests having multi-input CNNs that learn from paired features can enhance diagnostic accuracy of the CAD system.

To further support our method, an ablation study has been conducted to assess the accuracy of the proposed method. The study shows that the proposed fusion using multi-input CNN outperformed single-input frameworks. In that study, a single input CNN with the same structure was built and evaluated. Four scenarios were evaluated. Scenarios 1 and 2 use T2-weighted images and ADC maps, respectively. Scenario 3 uses a probability voting scheme between the prediction of scenarios 1 and 2. We used the following equation to acquire the resultant probability after voting: $P_{v}=\frac{1}{2}\left(P_{T2_{Weighted}}+P_{ADC}\right)$. Scenario 4 uses a single input that combines T2-weighted images and ADC maps in the input channels. Results obtained from the four scenarios are shown in Table 5. Using a multi-input CNN enhances the classification accuracy. The two-CNN voting scenario showed high specificity, but a low accuracy, sensitivity and dice coefficient compared to the proposed method. This ablation study suggests that having independent features for each input can enhance the detection performance of the CAD system.

The main focus of this study is to investigate the ability to extract the texture features associated with thyroid cancer by combining the texture in two input CNN with two independent branches. The network was designed to minimize the number of layers in order to extract the texture patterns that can be linked to the anatomical structure in the nodules. This optimized architecture also supports fast processing, which can enable further integration with MRI scanner devices to present the visual features automatically extracted from MRI images. As a follow-up step in our study to evaluate the heterogeneity of texture features between MRI modalities, we applied a method to extract and cluster the learned features for each modality. An illustration is presented in Figure 5a and the obtained feature visualization in each input is presented in Figure 5c,d. That visualization suggests a heterogeneity in texture patterns between MRI modalities and supports the use of our method for thyroid nodule classification.

Our system yielded promising results. However, there are some limitations that need to be addressed in order to go forward with further clinical trials. The number of samples is limited under the scope of our study, and the results can reflect the pattern that exists in this cohort. Our model needs to be applied to another cohort with a higher number of subjects in order to assess the homogeneity of texture across cohorts. More samples can be collected to sufficiently cover the full spectrum of thyroid cancer.

In total, this paper shows that extracting texture patterns using deep learning can improve the diagnostic performance and can help in performing accurate diagnosis of thyroid cancer. For future work, our experiments can be applied to bigger cohort. Additionally, our model can be adapted to perform classification of the types of thyroid cancer. It can be also adapted to perform staging of thyroid cancer. Other modalities can be added to the model to study the heterogeneity of MRI texture patterns in a more advanced way. Our model can also be adapted to study the texture patterns of thyroid tissues while using other imaging techniques such as US. Although, US can provide a limited capability of modeling thyroid cancer compared to MRI, having a model that combines US and MRI can contribute to establishing more accurate models to ensure precise and personalized medicine.

Data collection can be also expanded to collect multiple scan from each subject in a different time points. By doing this, we can study the correlation between DWI patterns and the patterns of the cell proliferation process, which is associated with thyroid nodules at different stages of thyroid cancer.

## Figures and Tables

**Table 1:** Summary of the network parameters used during model training.

Parameter	Value
Kernel Size	3 × 3 × 3
Number of Convolution Kernels	32
Number of 1 × 1 Kernels	16
Fully Connected Layers	2
Convolutional Layers	2
Activation	ReLU
Pooling Size	2 × 2 × 2
Pooling	MaxPooling
Number of Epochs	100
Input Shape	48 × 48 × 20

**Table 2:** Statistical analysis results for the Welch *t*-test on the pixel-level intensity variations between the malignant and benign groups.

	Welch Two-Sample *t*-Test		
MRI Parameter	CI Δmean	t	p
T2	−4% to −1%	−2.28	0.023
ADC500	5% to 9%	7.87	<0.001
ADC1000	26% to 34%	14.87	<0.001
ADC1500	4% to 8%	6.12	<0.001

**Table 3:** Comparative performance for the proposed multi-input CNN system and machine learning methods that use hand-crafted features. “DT”—Decision Tree. “RF”—Random Forest; “NB”—Naive Bayes; “SVM”—Support Vector Machine.

	Evaluation Metrics			
Method	Accuracy	Sensitivity	Specificity	Dice Coefficient
DT classifier	0.70	0.66	0.70	0.57
NB classifier	0.76	0.73	0.77	0.63
RF classifier	0.77	0.67	0.77	0.53
SVM classifier	0.56	0.40	0.73	0.48
Proposed Multi-Input CNN	0.87	0.69	0.97	0.79

**Table 4:** Comparative performance of the proposed multi-input CNN system with state-of-the-art CNN-based classification.

	Evaluation Metrics			
Method	Accuracy	Sensitivity	Specificity	Dice Coefficient
AlexNet	0.61	0.53	0.66	0.49
ResNet18	0.49	1.00	0.22	0.58
Proposed Multi-Input CNN	0.87	0.69	0.97	0.79

**Table 5:** Ablation study results for the proposed system.

	Evaluation Metrics			
Method	Accuracy	Sensitivity	Specificity	Dice Coefficient
Single-Input CNN (T2-Weighted only)	0.76	0.56	0.87	0.62
Single-Input CNN (ADC only)	0.72	0.63	0.77	0.61
Two-CNN voting (base-images + ADC)	0.83	0.63	0.93	0.71
Multi-Input CNN (Proposed Method)	0.87	0.69	0.97	0.79

## Data Availability

Requests made to the corresponding author will be addressed and the available data can be provided.
